# Neurological mechanism and efficacy of acupuncture for breast cancer-related insomnia: a study protocol for randomized clinical trial

**DOI:** 10.3389/fneur.2023.1278564

**Published:** 2023-12-22

**Authors:** Lumin Liu, Ping Yin, Yiyue Dong, Qian Fan, Yisheng Huai, Shijie Zhang, Shunyi Lv, Xueyang Wang, Yuelai Chen

**Affiliations:** LongHua Hospital Shanghai University of Traditional Chinese Medicine, Shanghai, China

**Keywords:** breast cancer, insomnia, acupuncture, fMRI, randomized controlled trial, protocol

## Abstract

**Background:**

Breast cancer survivors (BCSs) are at a higher risk of developing insomnia. The negative effects of cancer-related insomnia (CRI) include depression, anxiety, fatigue, aggressive pain, impaired immune functioning, decreased quality of life, and even increased cancer mortality. Although preliminary progress has been made in the treatment of CRI with acupuncture, the evidence is insufficient and the neurological mechanism underlying the effect of acupuncture is still unclear.

**Methods:**

The study employs a single-blinded, randomized, controlled trial design. A total of 80 participants will be randomly allocated in a 1:1 ratio to either the treatment group (*n* = 40) or the control group (*n* = 40). The former will receive acupuncture treatment, while the latter will receive sham acupuncture treatment. Both groups will receive 12 sessions over a 4-week period, three times per week (every other day), and each session will last for 30 min. Follow-up assessments will be conducted in week 8. The primary outcome will be the treatment response rate. Secondary outcomes include the change in Insomnia Severity Index (ISI), the treatment remission rate, actigraphy sleep assessment, Generalized Anxiety Disorder Scale (GAD-7), Patient Health Questionnaire-9 (PHQ-9), Quality of Life Core Scale (QLQ-C30), the weekly usage of remedial drugs, and functional magnetic resonance imaging (fMRI) analysis. Data for the outcomes will be collected at week 0 (the baseline), week 1 (the intervention period), week 4 (the post-treatment period), and week 8 (the follow-up period).

**Discussion:**

The objective of this study is to assess the efficacy of acupuncture for patients with CRI in comparison with sham acupuncture. Additionally, the research aims to explore the neuropathological mechanisms of CRI and provide the first evidence on the characteristics of acupuncture treatment using fMRI. We expect that the results of this study will provide valuable scientific evidence of acupuncture treatment for CRI.

**Clinical trial registration**: Chinese Clinical Trial Registry, identifier ChiCTR2300070349: https://www.chictr.org.cn/showproj.html?proj=188677.

## Background and objective

In recent times, breast cancer has overtaken lung cancer as the foremost cause of cancer incidence worldwide. According to the 2020 Global Cancer Observatory (GLOBOCAN) database, which covers 36 types of cancers in 185 countries, an estimated 2.3 million new cases of breast cancer have been reported. These cases account for approximately 11.7% of all cancer cases (1). While the survival rate for breast cancer survivors (BCSs) remains relatively high in most countries, it is imperative to address the significant issue of reduced life quality among them, especially cancer-related insomnia (CRI). Therefore, this particular concern has gained prominence as an important clinical priority.

A large proportion of BCSs, ranging from 53.5 to 79.6%, have experienced clinically elevated and persistent insomnia symptoms, both during and after completing treatment (2, 3). These symptoms encompass dissatisfaction with sleep quality or quantity and are characterized by difficulties of falling sleep, maintaining sleep, or experiencing early morning awakenings, which cause notable distress or impairment in daytime functioning (4). The situation may arise from psychological distress following a cancer diagnosis, concurrent cancer symptoms, and treatment-related side effects (5). Moreover, CRI further heightens the risk of psychological and physical morbidity, ultimately impacting the overall quality of life negatively (6, 7).

Presently, interventions for treating CRI encompass both pharmaceutical and non-pharmaceutical approaches. However, commonly employed sedative and hypnotic drugs, despite their rapid onset of action, often come with various sequelae, withdrawal effects, dependence, and addiction (8). Hence, there is significant value in exploring complementary and alternative therapies that offer effective outcomes with minimal side effects. A growing interest in acupuncture therapy has revealed its potential to enhance sleep quality while addressing mental and emotional distress symptoms (9). Nevertheless, the limited study designs of previous research diminish the persuasiveness of the evidence, and the underlying mechanism of acupuncture remains incompletely understood (10, 11).

Therefore, the purpose of this study is to examine the clinical efficacy and safety of acupuncture in treating CRI. Our investigation will comprehensively assess patients’ sleep through subjective and objective measures. Furthermore, due to the lack of functional imaging studies on CRI, this trial will incorporate functional magnetic resonance imaging (fMRI) to explore the potential neural mechanisms underlying the effectiveness of acupuncture in treating CRI.

## Methods

### Study design

**Figure 1:**
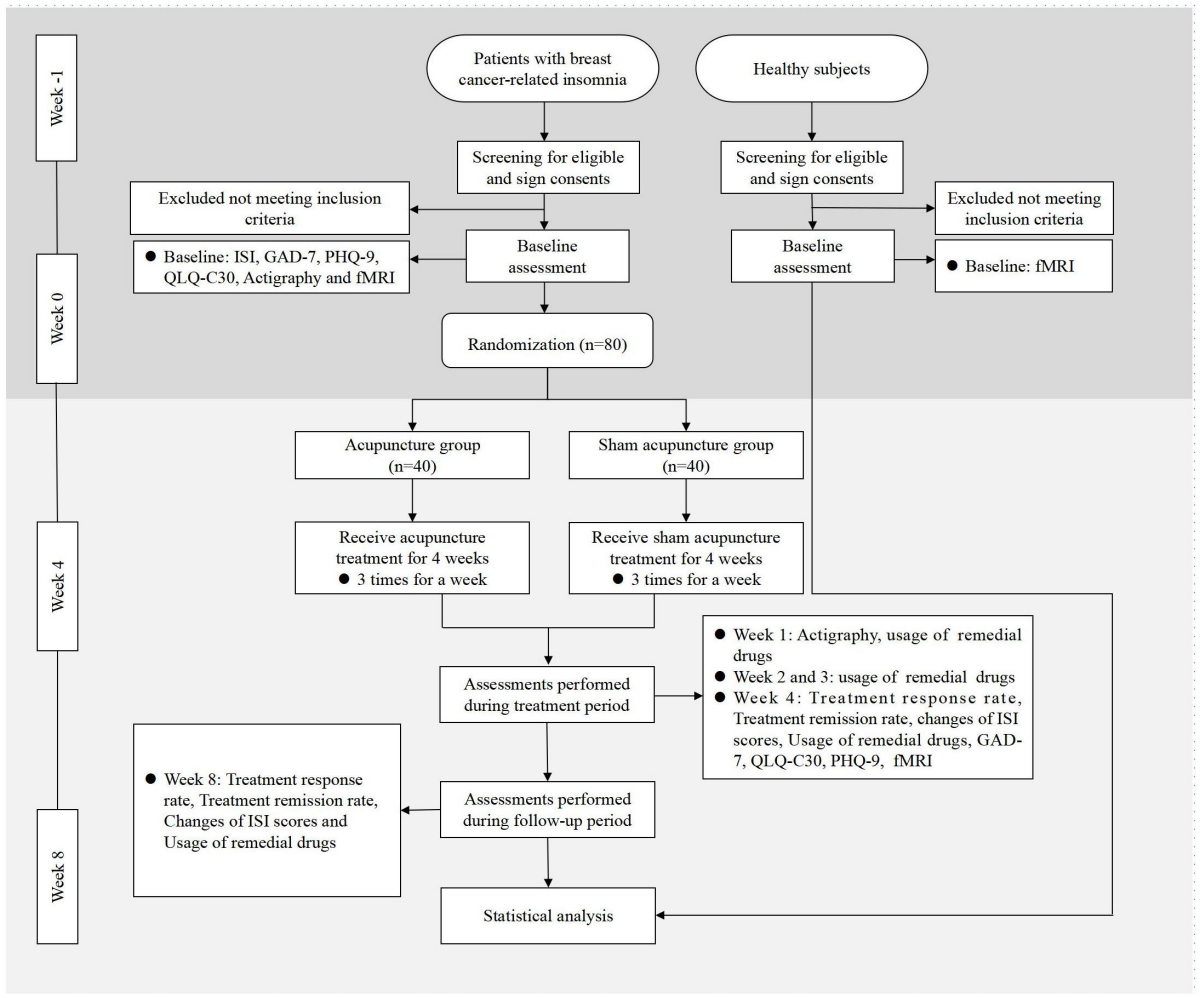
Flowchart of the study. ISI, Insomnia Severity Index; GAD-7, Generalized Anxiety Disorder Scale; PHQ-9, Patient Health Questionnaire-9; QLQ-C30, Quality of Life Core Scale; fMRI, Functional Magnetic Resonance Imaging.

As a randomized, single blinded, sham-controlled trial, this study has been registered in the Chinese Clinical Trial Registry (ChiCTR2300070349). The protocol is based on the Standard Protocol Items: Recommendations for Intervention Trials (SPIRIT) 2013 in Supplementary File 1. This study was approved by the Medical Ethics Committee of LongHua Hospital Shanghai University of Traditional Chinese Medicine (SHUTCM) (NO.2023-LHXS-009). Any modifications to the protocol will be reported to both the ChiCTR and the Medical Ethics Committee. The flowchart of the study is shown in Figure 1, while the schedule of enrollment, interventions, and assessment is shown in Table 1.

**Table 1:** Schedule of enrolment, interventions, and assessments.

Study period	Enrolment		Intervention period				Follow-up period
Time point	Week-1	Week 0	Week 1	Week 2	Week 3	Week 4	Week 8
Eligibility screening	**×**						
Sign informed consent	**×**						
Medical history	**×**						
Randomization		**×**					
*Intervention*							
Acupuncture			**×**	**×**	**×**	**×**	
Sham acupuncture			**×**	**×**	**×**	**×**	
*Primary outcomes*							
Treatment response rate						**×**	
*Secondary outcomes*							
Treatment remission rate						**×**	**×**
Treatment response rate							**×**
Changes of ISI scores from baseline		**×**				**×**	**×**
Actigraphy sleep assessment		**×**	**×**			**×**	
GAD-7		**×**				**×**	
PHQ-9		**×**				**×**	
QLQ-C30		**×**				**×**	
fMRI		**×**				**×**	
Adverse events			**×**	**×**	**×**	**×**	
Weekly usage of remedial drugs			**×**	**×**	**×**	**×**	**×**
*Success of blinding*						**×**	

ISI, Insomnia Severity Index; GAD-7, Generalized Anxiety Disorder Scale; PHQ-9, Patient Health Questionnaire-9; QLQ-C30, Quality of Life Core Scale; fMRI, Functional Magnetic Resonance Imaging.

### Patient recruitment

A total of 80 patients with breast cancer-related insomnia will be recruited from both hospital websites and outpatient clinics at LongHua Hospital SHUTCM. Patients will undergo eligibility assessment based on predefined inclusion and exclusion criteria. Those who meet the criteria will provide informed consent and be randomly allocated in a 1:1 ratio to either the treatment group or the control group. In addition, 10 healthy subjects will also be recruited for this trial. Participants will have the right to withdraw from the trial at any time, and their personal data will be used exclusively for medical research purposes.

### Criteria for inclusion

For CRI patients, the inclusion criteria are as follows:

1. Meet the diagnosis of stage I to III breast cancer and also meet the DSM-IV diagnostic criteria for insomnia, the symptoms of which occurred after breast cancer diagnosis and lasted for at least 1 month.
2. Aged 18 to 75 years, female.
3. Eastern cooperative oncology group performance status score ≤ 2.
4. Sleep severity index (ISI) score ≥ 8.
5. Survival prediction ≥6 months.
6. No prior acupuncture treatment.
7. Right-handed.
8. No MRI contraindications such as MR-incompatible metal implants or claustrophobia.
9. Consent to participate and provide written informed consent.

For healthy subjects, the inclusion criteria are as follows:

1. Age-, hand dominance-, and educational level matched to the selected CRI patients.
2. Self-reported good sleep quality with an ISI score of <8.
3. Absence of previous functional or organic disease or history of head trauma.
4. No MRI contraindications for the CRI patients.
5. Consent to participate and provide written informed consent.

### Criteria for exclusion

For CRI patients, the exclusion criteria are as follows:

1. Insomnia associated with cancer pain (Numeric Rating Scale for Pain ≥4) or other physical and mental illnesses.
2. Irregular sleep habits or night shift work.
3. History of drug abuse or addiction.
4. Patients who have taken sedative or hypnotic drugs over the past 2 weeks, accepted other treatment for insomnia over the past 3 months, or participated in other clinical trials over the last month.
5. Scheduled for radiotherapy, chemotherapy, or surgery during the trial.
6. Concomitant serious heart, liver, kidney, and other major diseases.
7. Pregnant or breastfeeding.
8. Inability to understand and complete the assessment scale.
9. Ulcers, abscesses, and infections at the needling site.

For healthy subjects, the exclusion criteria are as follows:

1. Pregnant or breastfeeding.
2. Irregular sleep habits or night shift work.

### Randomization, allocation concealment, and blinding

Randomization will be performed using random numbers generated by a professional statistician with SAS version 9.4 (SAS Institute Inc., Cary, NC, United States). An assistant will open an opaque envelope containing a random distribution card in the order matching the hospital visits to assign patients randomly to the treatment or control group. To ensure blinding, researchers will receive training and adhere to departmental separation principles. While blinding the acupuncturist is not possible, patients, outcome assessors, MRI scanner operators, and data analysts will be blinded. Patients will be scheduled at different times and placed in separate rooms to prevent interaction. A black patch will be worn during treatment to conceal acupoint locations and manipulation. Additionally, an acupuncture device will facilitate successful blinding. A blinding assessment will be conducted after treatment with the question: “Which intervention do you think you received, acupuncture treatment, sham acupuncture treatment, or uncertain?”

## Interventions

### Treatment group

**Figure 2:**
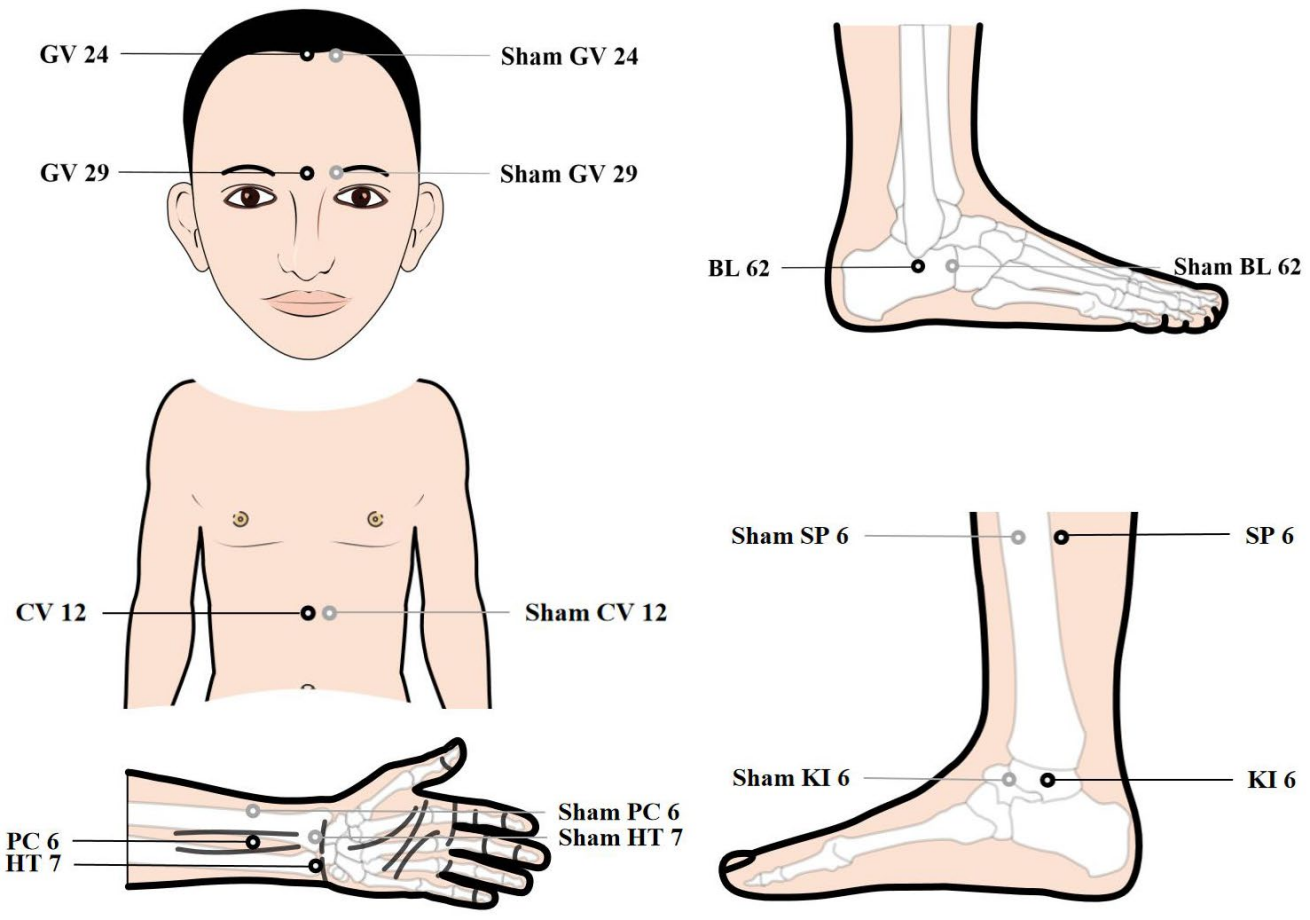
Image of acupoints and control acupoints.

**Figure 3:**
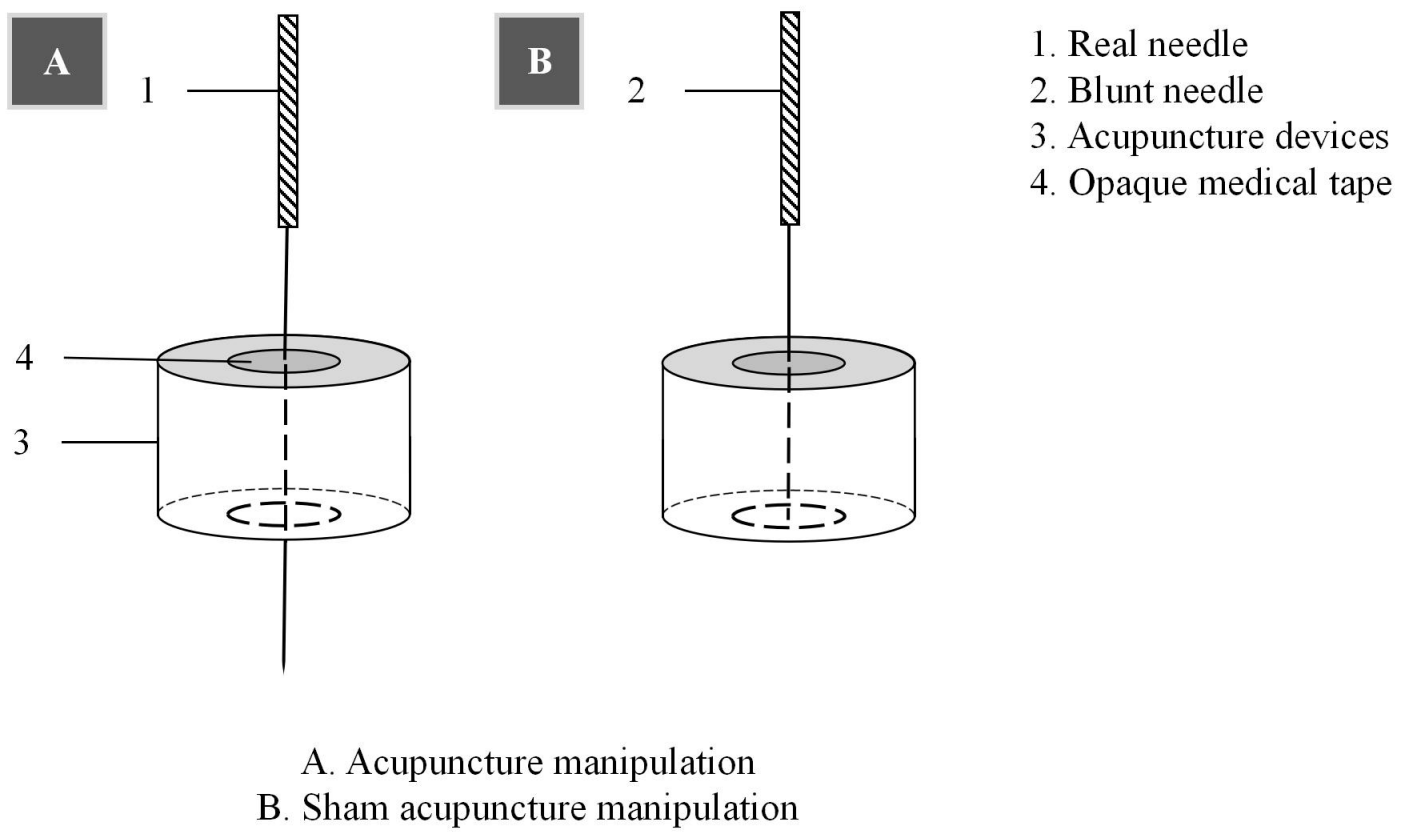
Acupuncture and sham acupuncture manipulation.

Patients will be treated with a supine position at unilateral Shen Ting (GV 24), Yin Tang (GV 29), Zhong Wan (CV 12) and bilateral Nei Guan (PC 6), Shen Men (HT 7), San Yin Jiao (SP 6), Zhao Hai (KI 6), and Shen Mai (BL 62). Locations are determined according to the National Standard of People’s Republic of China (GB/T12346-2010) is shown in Figure 2, and the specific manipulating techniques are described in Table 2. In this group, sterile acupuncture needles from Suzhou Medical Supplies Factory Co.’s Huatuo (size 0.25*25 mm and 0.25*40 mm) will be used. Prior to needle insertion, routine disinfection will be performed, and acupuncture devices will be applied to each location. The acupuncture device is a hollow patch covered with an opaque medical tape on the top, adhering to the skin at the base (shown in Figure 3). For the acupuncture group, needles will be pierced through the patch into the skin and then manipulated by insertion, lifting and twisting to activate the patients’ sensation of “de qi,” characterized by sensations of swelling, soreness, numbness, and heaviness. Participants will receive three treatment sessions per week (every other day), each lasting for 30 min, for a total of 12 sessions over a 4-week period. Follow-up assessments will be conducted in week 8.

**Table 2:** Locations and manipulations of acupoints in the intervention group.

Acupoint	Location	Manipulation
Nei Guan (PC 6)	On the anterior aspect of the forearm, between the palmaris longus and flexor carpi radialis tendons and 2 *cun* proximal to the palmar wrist crease	Insert the needle perpendicularly for 0.5–1.0 *cun*
Shen Men (HT 7)	On the anterior and medial aspect of the wrist, radial to the flexor carpi ulnaris tendon at the palmar wrist crease	Insert the needle perpendicularly for 0.3–0.5 *cun*
Shen Ting (GV 24)	On the anterior midline of the head. This acupoint is found 0.5 *cun* superior to the anterior hairline	Insert the needle transversely for 0.5–0.8 *cun*
Yin Tang (GV 29)	At the midpoint between the medical ends of the eyebrows	Insert the needle transversely for 0.3–0.5 *cun*
Zhong Wan (CV 12)	On the anterior midline of the upper abdomen, 4 *cun* above the umbilicus	Insert the needle perpendicularly for 0.8–1.2 *cun*
Zhao Hai (KI 6)	On the medial aspect of the foot, in the depression inferior to the medial malleolus and 1 *cun* inferior to the prominence of the medial malleolus	Insert the needle perpendicularly for 0.3–0.5 *cun*
San Yin Jiao (SP 6)	On the tibial aspect of the leg, posterior to the medial border of the tibia and 3 *cun* superior to the prominence of the medical malleolus	Insert the needle perpendicularly for 1.0–1.5 *cun*
Shen Mai (BL 62)	On the lateral aspect of the foot, this acupoint is located directly inferior to the prominence of the lateral malleolus, in the depression between the inferior border of the lateral malleolus and the calcaneus	Insert the needle perpendicularly for 0.3–0.5 *cun*

### Control group

Control acupoints will be used in this group, which are 1 cun laterally from the acupoints in the treatment group (shown in Figure 2), as we applied in previous research studies (12). Blunt needles with flat tips were selected for this study. After routine disinfection, acupuncture devices will be applied to each location. When the blunt needle tip penetrates the patch and touches the skin, the patient will experience a pricking sensation; however, no actual needle is inserted into the skin. With the patch, the needles are visually perceived as being inserted into the skin, mirroring the appearance of the treatment group. However, the acupuncturist will press the acupoints with a dry cotton ball to let the patient feel the ‘needles’ being withdrawn after treatment. Furthermore, the control group adheres to the same duration of needle retention, treatment time, and follow-up time as the treatment group.

Patients in both groups will receive sleep hygiene instructions, including recommendations to avoid consuming coffee, tea, or alcohol, maintain a regular sleep habit, create a quiet sleep environment, and promote a positive mood. If they experience severe discomfort caused by insomnia for two or more consecutive days, they are allowed to take hypnotic medications under the doctor’s guidance. However, the name of the medication, the doses, the date of administration, and the reactions to the medication will need to be recorded in detail on the case report form.

### fMRI examination

fMRI scans will be performed with an Ingenia 3.0 Tesla MRI scanner (Philips Medical Systems, Best, Netherlands) in the Department of Radiology at LongHua Hospital SHUTCM. During scanning, the participants are supine, with their heads immobilized and their ears plugged to minimize head movement and gradient noise interference. They will be instructed to wear black patches, close eyes, and remain still and awake throughout the process. Furthermore, the fMRI scans will be conducted by the same technician (WXY) using the same machine, and two qualified radiologists (LSY and ZSJ) will review the images for quality and procedure compliance after each scan, including the detection of any brain lesions or structural abnormalities. Both the technician and the radiologist have expertise in MRI imaging.

T1-weighted structural images are acquired with the following parameters: repetition time (TR) = 8.2 ms, echo time (TE) = 3.2 ms, slice thickness = 1.00 mm, slice number = 192, field of view (FOV) = 256 × 256 mm, flip angle = 8°, matrix size = 256 × 256 mm for standard space co-registration. Then the resting-state data are acquired as follows: repetition time (TR) = 2,000 ms, echo time (TE) = 30 ms, slice thickness = 3.5 mm, slice number = 33, field of view (FOV) = 224 × 224 mm, flip angle = 90°, and matrix size = 64 × 64 mm to obtain fMRI data. Scanning will be terminated if the participants feel any discomfort.

### Outcomes

The primary outcome measure is the treatment response rate, defined as the percentage of individuals whose total ISI score is decreased by ≥8 points from baseline at the end of treatment (week 4) (13).

ISI is a subjective and validated measure assessing various aspects of sleep, including sleep onset, maintenance, early awakening, dissatisfaction, interference with daytime functioning, life quality, and mental distress. The seven-item questionnaire uses a 0–4 scale, yielding a total score range of 0–28. Scores correspond to absence (0–7), mild (8–14), moderate (15–21), or severe (22–28) insomnia. The ISI is a concise, reliable tool commonly employed in general practice, sensitive to treatment response (14).

The secondary outcomes include the following eight items:

1. The change in ISI scores.
2. The treatment remission rate: the percentage of people with ISI < 8.
3. Actigraphy sleep assessment: It is a watch-shaped device to objectively record sleep patterns. It includes measurements of total sleep duration (TST), sleep–wake duration (WASO), and sleep efficiency (SE). Consequently, actigraphy serves as an effective tool for evaluating sleep quality in cancer patients (15).
4. Generalized Anxiety Disorder Scale (GAD-7): It is a concise anxiety self-assessment instrument consisting of seven items scored on a 0–3 scale, yielding a total score range of 0–21. Scores of 5–9 indicate mild anxiety, 10–14 suggest moderate anxiety, and 15–21 represent severe anxiety (16).
5. Patient Health Questionnaire-9 (PHQ-9): This nine-item validated depression self-assessment tool scored on a 0–3 scale, with a total score range of 0–27. Interpretation of scores categorizes depression as mild (5–9), moderate (10–14), severe (15–19), or very severe (20–27) (17).
6. Quality of Life Core Scale (QLQ-C30): This 30-item questionnaire evaluates the QoL among cancer survivors. It comprises functional scales (physical, role, cognitive, emotional, and social functioning), a global QoL scale, symptom scales (fatigue, nausea and vomiting, and pain), and single items (appetite loss, diarrhea, dyspnea, constipation, insomnia, and financial impact) (18).
7. The weekly usage of remedial drugs: The percentage of participants who used hypnotic medications.
8. fMRI: The fMRI data will be pre-processed and analyzed using SPM8 software platform (SPM8, Wellcome Department of Imaging Neuroscience, London, United Kingdom) with MATLAB 2016b (MathWorks, Inc., Natick, MA, United States). Images will be pre-processed, including artifact correction (AFNI and RETRO-ICOR), head motion correction (FSL and MCFLIRT), and brain extraction (FSL and BET). Cortical surface reconstruction with boundary-based registration (FreeSurfer and bbregister) enhances structural–functional co-registration. After spatial smoothing and high-pass temporal filtering, various analytical methods (ALFF, ReHo, and seed-based FC) will assess neural response.

Outcomes will be measured during four different time points: week 0 (the baseline), week 1 (the intervention period), week 4 (the post-treatment period), and week 8 (the follow-up period). At week 0, ISI scores, actigraphy, GAD-7, PHQ-9, and QLQ-C30 will be collected. At week 4, the primary and all secondary outcomes will be tested. At week 1, actigraphy will be measured to observe the short-term effect of treatment. At week 8, the primary and a few secondary outcomes (i.e., the change of ISI scores, the treatment remission rate, and the weekly usage of remedial drugs) will be recorded. Additionally, at week 0 and week 4, neuroimaging data will be acquired to test the neural effects of the treatments for CRI patients, while the healthy subjects only need to be scanned once at week 0 (the baseline). Moreover, weekly usage of remedial drugs will be measured from week 1 to week 8.

### Safety evaluation

Treatment-associated adverse events (AEs) comprise dizziness, hematoma, broken needles, and infection. Researchers will document AEs in the CRF, noting dates, severity, relevance, and resolution. Proficient acupuncturists, well-versed in acupoint localization, precise manipulation, and aseptic procedures, along with the use of high-quality, disposable sterile needles, can effectively mitigate AEs. Patients’ preparation, ensuring they are neither hungry nor fatigued, further contributes to AE avoidance. In the occurrence of any observed AEs, acupuncturists will promptly evaluate their severity and undertake appropriate measures. These may encompass discontinuation of treatment, provision of rest post-dizziness, application of gentle pressure to mitigate bleeding and hematoma formation, or referral of the patient to a surgeon or physician for instances involving needle breakage or infection. Serious adverse events will be reported promptly to the safety committee and actively managed. Meanwhile, details of the termination of treatment will be recorded, including the final time and outcome, to aid in the statistical analysis of AEs.

### Sample size calculation

Sample size calculation utilized the ‘Test For Two Proportions (Test Version)’ module of PASS software (version 15.0.5, NCSS, LLC) with a two-tailed α of 0.05, power of 80%, and a 1:1 ratio. Based on our preliminary pilot results, the treatment response rate at week 4 was 33.3% (5/15) in the treatment group and 6.7% (1/15) in the control group, with an effect size of 0.71. The sample size encompasses 32 participants allocated to each group. Assuming a 20% dropout rate, the required sample size is 40 participants per group, totaling 80 participants.

### Statistical methods

Statistical analysis will employ SAS software for the full analysis set, per-protocol set, and safety set. Missing values will be handled using the last observation carryover method. Continuous variables will be presented as mean ± SD (normal distribution) or median (range) (non-normal distribution) and assessed with the t-test or Mann–Whitney U test. Categorical variables will be presented as frequencies or percentages and analyzed using chi-square or Fisher’s exact tests. CIs will be set at 95% with a significance level of 5% (*p* < 0.05). To avoid multiplicity, we will use a Bonferroni adjustment for multiple comparisons of fMRI among the treatment group, control group, and healthy subjects. With three groups (k = 3), the adjusted test level (α’) was set at 0.0167 (α/3 = 0.05/3). Pearson’s correlation will examine the association between changes in brain activity and clinical outcome improvement within each group. The success of blinding will be assessed using the Bang’s Index. In addition, to mitigate the influence of age and other confounding factors, we will employ covariance analysis on the continuous data.

### Quality control, data management, and monitoring

Prior to treatment, licensed acupuncturists with a minimum of 5 years’ clinical experience will receive rigorous standardized training in acupoint locations, manipulation techniques, and masking application for acupuncture and sham acupuncture. Researchers will receive standardized training on the study protocol, Standard Operating Procedures, and scale evaluation to enhance research validity. A detailed data management strategy will be created, comprising data collection, entry, and management. To ensure accuracy, data will be entered independently by two researchers, and then data will be confirmed by both two researchers. Data quality and research progress will be checked regularly by research assistants and supervised by monitors. The monitoring committee comprises individuals well versed in clinical practice, research methodologies, and statistics. It operates independently from the study team, maintaining no direct involvement in the trial’s execution. Their responsibilities encompass overseeing trial conduct, decision-making processes, and ensuring the integrity and safety of the gathered data every 6 months.

## Discussion

Acupuncture has been utilized in the treatment of insomnia in China since ancient times. Previous single-group or pilot trials have demonstrated that acupuncture treatment, either alone or in combination with auricular acupressure, can be advantageous for improving sleep quality (10, 11) among cancer survivors. The efficiency of acupuncture surpasses fluoxetine or tamoxifen in improving insomnia symptoms and reducing cancer-related mental disorders (19, 20). Notably, the comparisons with positive drugs should be interpreted with caution as these agents are not specifically targeted for sleep-related conditions. Moreover, studies have revealed that acupuncture treatment can improve sleep onset latency, total sleep time, and sleep efficiency; however, compared to sham control, acupuncture does not exhibit a significant difference in reducing ISI (21). Therefore, the confirmation of acupuncture’s superiority over sham control remains unresolved. In summary, the paucity of studies exploring curative effects necessitates a blinded randomized clinical trial to validate existing findings. The mechanisms of acupuncture in treating CRI are still unknown.

Abnormal activity has been observed in multiple brain regions, including the prefrontal lobe, temporal lobe, parietal lobule, anterior cingulate, supramarginal gyrus, and precuneus among victims of insomnia. Remarkably, acupuncture has demonstrated a significant improvement in symptoms of insomnia and the ability to modulate brain activity in these regions (22–24). Consequently, we propose that acupuncture may also ameliorate CRI by influencing the activity of correlated brain regions. To investigate this, we have designed a randomized controlled trial to assess the effects of acupuncture on CRI, with a specific focus on its impact on the hyperarousal state of the brain, as measured by fMRI methods.

To enhance result reliability and minimize bias, we will incorporate the following quality control measures. First, both real and sham acupuncture treatments will employ identical auxiliary device which is granted as a patent (ZL 2023 20605629.3). It is visually indistinguishable to fulfill blinding requirements, encourage better compliance of patients, and be capable of penetrating adhesive tape to simulate the sensation of needle insertion. Previous clinical studies have validated this manipulation of placebo needles as feasible (12). Second, based on prior research studies revealing distinct brain activity patterns in left-handed and right-handed individuals (25), this study exclusively recruits right-handed participants due to the considerably higher prevalence of right-handedness in China. Third, a comprehensive evaluation of patients’ sleep quality will be conducted using both objective and subjective measures, including the widely recognized ISI and actigraphy sleep assessment. These combined approaches will provide a more comprehensive understanding of patients’ sleep patterns.

However, there are limitations to the current study design. This trial is not conducted as a multi-center study, which may raise concerns about the generalizability of the results to a broader population. In light of the inherent characteristics of acupuncture interventions, the implementation of a double-blind approach is not feasible.

## Conclusion

This research endeavors to assess the effectiveness of acupuncture for patients with CRI when contrasted with sham acupuncture. Additionally, our study seeks to delve into the underlying neuropathological mechanisms of CRI while employing fMRI to investigate the distinct neural response characteristics elicited by acupuncture treatment.

## Ethics statement

The studies involving humans were approved by the Medical Ethics Committee of LongHua Hospital Shanghai University of Traditional Chinese Medicine. The studies were conducted in accordance with the local legislation and institutional requirements. The participants provided their written informed consent to participate in this study.

## Author contributions

LL: Methodology, Writing – original draft. PY: Methodology, Writing – original draft. YD: Methodology, Writing – review & editing. QF: Methodology, Writing – review & editing. YH: Formal analysis, Writing – review & editing. SZ: Methodology, Writing – review & editing. SL: Methodology, Writing – review & editing. XW: Methodology, Writing – review & editing. YC: Conceptualization, Funding acquisition, Supervision, Writing – review & editing.
